# Decreased Substrate Stiffness Promotes a Hypofibrotic Phenotype in Cardiac Fibroblasts

**DOI:** 10.3390/ijms22126231

**Published:** 2021-06-09

**Authors:** Rachel C. Childers, Pamela A. Lucchesi, Keith J. Gooch

**Affiliations:** 1 Department of Biomedical Engineering, The Ohio State University, Columbus, OH 43210, USA; childers.73@osu.edu; 2 Department of Pharmacology, New York Medical College, Valhalla, NY 10595, USA

**Keywords:** cardiac fibroblast, stiffness, volume overload

## Abstract

A hypofibrotic phenotype has been observed in cardiac fibroblasts (CFs) isolated from a volume overload heart failure model, aortocaval fistula (ACF). This paradoxical phenotype results in decreased ECM synthesis despite increased TGF-β presence. Since ACF results in decreased tissue stiffness relative to control (sham) hearts, this study investigates whether the effects of substrate stiffness could account for the observed hypofibrotic phenotype in CFs isolated from ACF. CFs isolated from ACF and sham hearts were plated on polyacrylamide gels of a range of stiffness (2 kPa to 50 kPa). Markers related to cytoskeletal and fibrotic proteins were measured. Aspects of the hypofibrotic phenotype observed in ACF CFs were recapitulated by sham CFs on soft substrates. For instance, sham CFs on the softest gels compared to ACF CFs on the stiffest gels results in similar CTGF (0.80 vs. 0.76) and transgelin (0.44 vs. 0.57) mRNA expression. The changes due to stiffness may be explained by the observed decreased nuclear translocation of transcriptional regulators, MRTF-A and YAP. ACF CFs appear to have a mechanical memory of a softer environment, supported by a hypofibrotic phenotype overall compared to sham with less YAP detected in the nucleus, and less CTGF and transgelin on all stiffnesses.

## 1. Introduction

Cardiac fibroblasts (CFs) play a crucial role in the physiological maintenance of extracellular matrix (ECM) and in many pathologies of the heart including myocardial infarction, hypertension, and cardiomyopathy. CFs are often suggested as a target for therapeutic strategies with most studies identifying cardiac myofibroblasts, an activated and profibrotic phenotype of CFs, as the main target in heart disease where there is fibrosis [1,2,3,4]. Therefore, most studies looking at CFs are framed in the context of the fibroblast-to-myofibroblast transition and emphasize strategies to make CFs less fibrotic. In these studies, quiescent fibroblasts become proto-myofibroblasts with increased cytoskeletal tension and then are activated into myofibroblasts with the addition of TGF-β [5,6].

In contrast to their role in fibrosis, fibroblasts also play a role in pathologies where there is insufficient ECM such as in emphysema [7], rheumatoid arthritis [8], and in volume overload (VO)-induced heart failure (HF) [9]. In VO HF, there is a net decrease in extracellular matrix in the left ventricle (LV) [10,11,12]. This net decrease in ECM within the tissue may affect the structural integrity of the chamber and disease progression. In chronic mitral regurgitation, one model of VO HF, there is a decrease in LV interstitial collagen content, downregulation of several profibrotic factors (i.e., CTGF and genes related to TGF-β signaling pathway) and extracellular matrix genes, as well as an increase in matrix metalloproteinases [13]. Similarly, in another model of VO HF, aortocaval fistula (ACF), there is a decrease in collagen content in the LV, increased expression of matrix metalloproteinases and decreased expression of pro-fibrotic factors [9,10]. The CFs isolated from ACF hearts exhibit what we called a “hypofibrotic” phenotype—that is, fibroblasts that exhibited markers of reduced ECM deposition and increased ECM degradation despite elevated levels of the pro-fibrotic factor TGF-β [14]. More specifically, CFs from VO hearts exhibited reduced secretion of connective tissue growth factor (CTGF), reduced levels of αSMA, and increased MMP-13 expression relative to CF isolated from control hearts [14]. Notably, these hypofibrotic fibroblasts (1) are isolated from ACF hearts that have elevated levels of the profibrotic factors TGF-β relative to control hearts, (2) secrete more TGF-β in culture than CF from control hearts, and (3) fail to exhibit the increased collagen type-1 secretion in culture in response to exogenous TGF-β seen in control CF [14].

Why CFs in VO hearts adopt this hypofibrotic phenotype is not known but we reasoned that changes in matrix stiffness might play a role. We recently reported the stiffness of the heart in the ACF model of VO decreases relative to control hearts as evidenced by altered pressure–volume loops in vivo and stress–stress curves from in vitro biaxial mechanical testing [15]. Matrix stiffness is an important regulator of many cellular functions including survival, proliferation, differentiation, and migration [16]. In fibroblasts, increased substrate stiffness supports transition to myofibroblasts, increases matrix deposition, and has been suggested to play roles in fibrosis in various tissues [5,17] including the heart [2,4]. In CFs isolated from control hearts, culture on relatively stiff substrates supports the formation of myofibroblasts. In contrast, very little is known about the effects of decreased substrate stiffness on fibroblast phenotype and matrix production but we reasoned that lowering stiffness below normal levels could promote the formation of hypofibrotic fibroblasts and reduced matrix production. In support of this notion, others showed that dermal fibroblasts in soft collagen gels were less responsive to TGF-β [18].

In addition to suggesting a potential reason the CFs from the less stiff VO hearts might be hypofibrotic, a stiffness perspective provides some insights into potential mechanisms. Several transcriptional regulators link alterations in cytoskeletal properties associated with changes in substrate stiffness to changes in gene expression. Myocardin-related transcription factor A (MRTF-A) transduces mechanical stress via the polymerization state of the actin cytoskeleton, where it is sequestered by G-actin and freed after polymerization to F-actin, which allows it to translocate to the nucleus. In the nucleus, MRTF-A promotes a fibrogenic program, including expression of αSMA and transgelin. On stiff (~20 kPa) polyacrylamide gels human lung fibroblasts increased F-actin and displayed an increase in MRTF-a nuclear translocation and a resulting increase in αSMA compared to soft gels (~0.5 kPa) [19].

Another transcriptional factor regulated by stiffness is yes-associated protein (YAP), which translocates to the nucleus through widened nuclear pores due to increased cytoskeletal tension or changes in cell shape [20,21]. YAP nuclear translocation promotes connective tissue growth factor (CTGF) and transgelin expression and has been associated with the myofibroblast activation of mesenchymal stromal cells [22], lung fibroblasts [23], and cancer-associated fibroblasts [24]. In pulmonary fibrosis, stiffness may play a role in regulating fibroblast phenotype as YAP nuclear translocation on stiff substrates is linked with fibroblast activation [20,23].

Due to the decreased tissue stiffness in ACF hearts and evidence showing stiffness influences fibroblast phenotype via several different transduction pathways, we reasoned that the decreased tissue modulus in ACF may play a role in the hypofibrotic phenotype of CFs. Here we test the hypothesis that (1) sham (control) CFs cultured on softer substrates would have a more hypofibrotic phenotype (with less MRTF-A and YAP in the nucleus and lower profibrotic molecules such as αSMA and CTGF), and conversely, (2) ACF CFs cultured on a stiffer substrate would shift toward a normal phenotype similar to sham CFs. To begin to characterize potentially relevant pathways, we assessed features related to the actin cytoskeleton and the transcriptional factors MRTF-A and YAP.

## 2. Results

### 2.1. Soft Stiffness Promotes Hypofibrotic Phenotype

**Figure 1:**
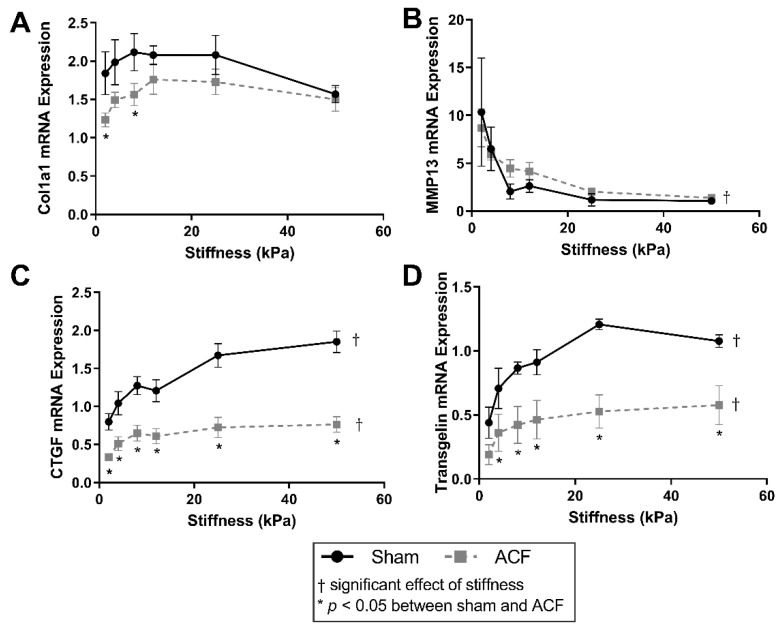
Soft substrates promote mRNA expression representative of a hypofibrotic phenotype. (**A**) Type-1 collagen mRNA expression is significantly different on softer substrates between sham and ACF. (**B**) MMP-13 mRNA expression decreases with increasing substrate stiffness in both sham and ACF CFs. (**C**,**D**) Relative mRNA expression of CTGF and transgelin follow a similar pattern, with relatively less in ACF compared to sham. * indicates *p* < 0.05 when comparing sham vs. ACF. † indicates a significant effect of substrate stiffness.

To better characterize the fibrotic potential of the sham (control) and ACF CFs on a range of stiffness, α-1 type 1 collagen (col1α1), MMP13, CTGF, and transgelin mRNA targets were measured (Figure 1). Overall, col1α1 expression peaks around 12 kPa for both sham and ACF CFs (Figure 1A). The expression of col1α1 was ~30% lower in ACF CFs on soft 2 and 8 kPa gels (*p* = 0.029 and *p* = 0.046, respectively) compared to sham. Expression of the collagenase MMP13 had an inverse relationship with stiffness in both sham and ACF CFs (Figure 1B). MMP13 expression decreased 90% for sham and 84% for ACF CFs between 2 and 50 kPa (*p* = 0.0011 and *p* = 0.0072, respectively). However, there was no significant difference between sham and ACF expression of MMP13 (*p* = 0.75). CTGF, a pro-fibrotic factor, was lower in ACF CFs compared to sham (*p* < 0.001) (Figure 1C). Increasing stiffness from 2 kPa to 50 kPa caused a 132% increase in CTGF in sham CFs (*p* < 0.001). The effect of stiffness on CTGF expression leveled off around 8 kPa for ACF CFs with an 128% increase between 2 and 50 kPa (*p* = 0.035). For all stiffness values, ACF’s transgelin expression was ~50% lower than expression in sham CFs (Figure 1D). Transgelin increased expression in both sham and ACF CFs with increasing stiffness (145% increase in sham and 205% in ACF between 2 and 50 kPa) (Figure 1D).

### 2.2. Increasing Stiffness Causes an Increase in the F/G-Actin Ratios

**Figure 2:**
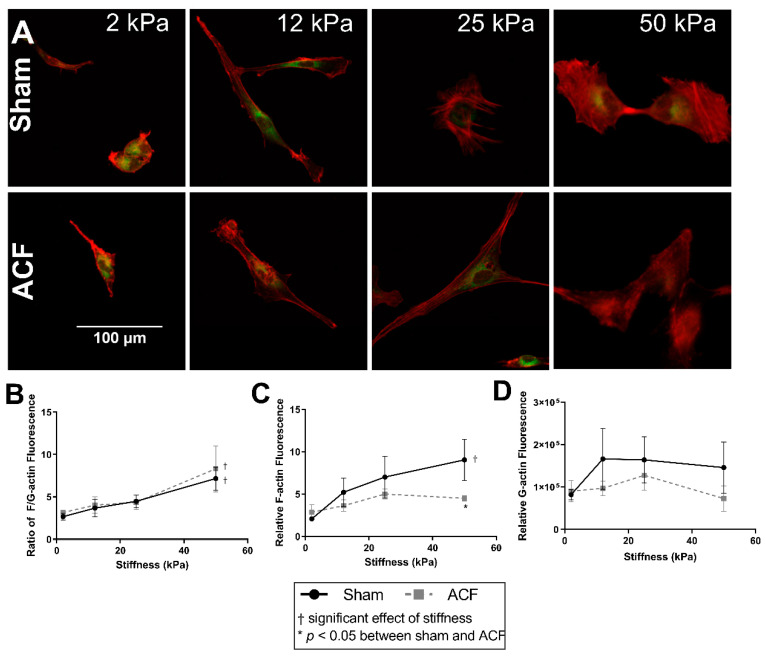
Increasing stiffness causes an increase in the F-actin-to-G-actin ratio in CFs from both sham and ACF as measured by fluorescent image analysis. (**A**) Sham (top row) and ACF (bottom row) CFs plated on polyacrylamide gels of a range of stiffness and stained with F-actin (red) and G-actin (green). (**B**) Quantification of the ratio of relative F-actin and G-actin fluorescence. (**C**) Relative F-actin fluorescence normalized to cells on a range of stiffness. (**D**) Relative G-actin fluorescence normalized to cells on a range of stiffness. * indicates *p* < 0.05 when comparing sham vs. ACF. † indicates a significant effect of substrate stiffness.

Since changes in the actin cytoskeleton are implicated in the hypofibrotic phenotype [14], we characterized the impacts of substrate stiffness on actin levels and distribution. Both cell spreading and the prominence of F-actin fibers increase with increasing substrate stiffness (Figure 2A). F-actin fluorescence increases with stiffness in sham but does not change significantly with ACF CFs (Figure 2C, *p* < 0.001 and *p* = 0.31, between 2 and 50 kPa gels). On 50 kPa gels, F-actin fluorescence was significantly higher in sham CFs compared to ACF CFs (*p* = 0.041, Figure 1C). Consistent with the increased prominence of F-actin fibers, the ratio of F-actin to G-actin increases with increasing stiffness in both sham and ACF (Figure 1C, *p* < 0.001). G-actin fluorescence was not significantly different with stiffness (*p* = 0.33) or between sham and ACF (*p* = 0.39, Figure 2D). Fluorescent staining technique did not reveal any difference between sham and ACF F/G-actin ratios (*p* = 0.79).

**Figure 3:**
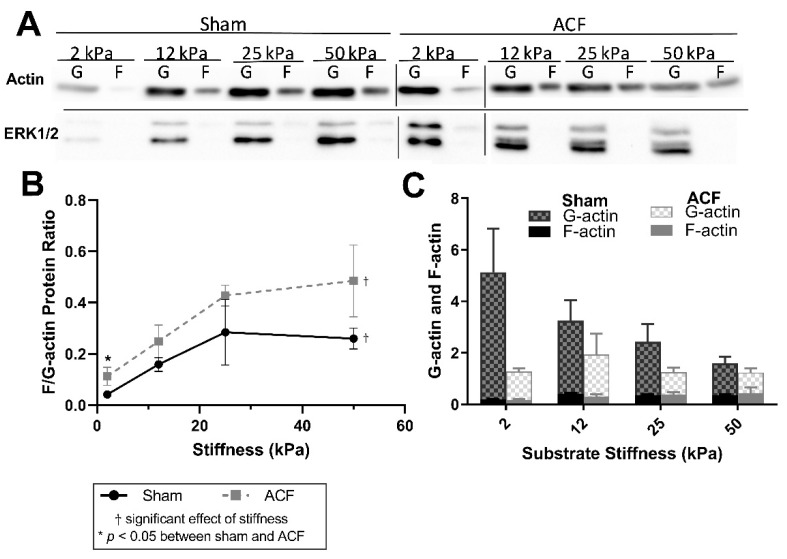
(**A**) Representative immunoblots for each condition. Bars indicate separate blots. (**B**) Immunoblot analysis confirms an increase in the F-actin-to-G-actin ratio with increasing stiffness. (**B**) F-actin-to-G-actin protein ratio measured from immunoblots. (**C**) Quantification of immunoblot densitometry, normalized to the ERK1/2 housekeeping proteins. * indicates *p* < 0.05 when comparing sham vs. ACF. † indicates a significant effect of substrate stiffness.

Using an immunoblot method (Figure 3A) to quantify F-actin and G-actin (Figure 3B), the results generally agreed fluorescent data. Comparing the ratios of F-actin and G-actin, data show an increase in the F/G-actin ratio with increasing stiffness (Figure 3B, *p* < 0.001). There were consistently larger F/G-actin ratios in ACFs compared to shams (Figure 3B, *p* = 0.027) with post-hoc analysis showing the only statistically significant difference between sham and ACF occurring on 2 kPa gels (*p* = 0.0004). It should be noted that only the ratios, rather than amounts, of G-actin and F-actin of a sample can be compared on the immunoblots, since the protocol for the commercial assay for F/G-actin ratios does not allow for a lane loading control (Figure 3A). However, normalizing to ERK1/2 housekeeping proteins reveals that the stiffness-induced increase in F/G-actin ratio in shams may be primarily due to a decrease in the total amount of G-actin (Figure 3C). In addition, the normalized amounts of total G-actin indicate that there may be an increased reservoir of G-actin in the sham CFs compared to ACF (*p* = 0.0069). 

### 2.3. αSMA Is Decreased in ACF CFs and at Lower Stiffness

**Figure 4:**
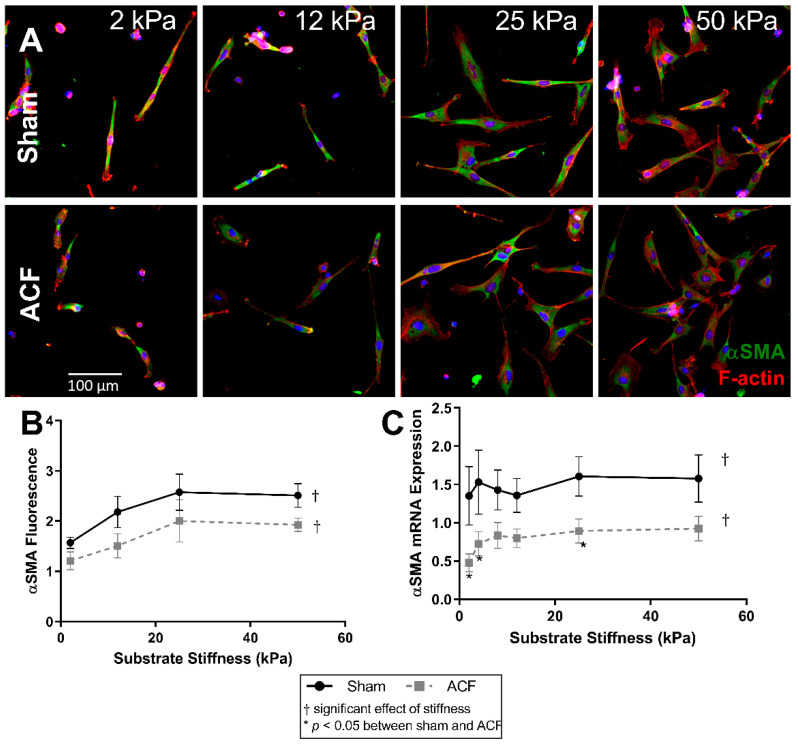
F-actin (red) and αSMA (green) fluorescent staining of sham and ACF (**A**,**B**) on different substrate stiffness. (**C**) αSMA mRNA expression increases with stiffness for both ACF and sham CFs, but ACF is generally lower overall. * indicates *p* < 0.05 when comparing sham vs. ACF. † indicates a significant effect of substrate stiffness.

Since αSMA is a marker of fibroblast phenotype and is downregulated in ACF CFs, we explored the relationship between αSMA and stiffness. The amounts of αSMA fluorescence increased with stiffness in both ACF and sham controls CFs (Figure 4A). αSMA fluorescence increased by similar amounts with stiffness in both sham and ACF CFs (e.g., 64% for sham, *p* = 0.0051, and 65% for ACF CFs, *p* = 0.022, between 2 and 25 kPa) (Figure 4A,B). There were no myofibroblasts, as defined by cells with αSMA positive stress fibers in any condition. However, there were proto-myofibroblasts, with increasing F-actin stress fibers with increased stiffness. The effect of stiffness on αSMA fluorescence plateaued at 25 kPa for both sham and ACF CFs. While the difference between sham and ACF did not reach significance using the fluorescent measurements (*p* = 0.085), there was a significantly lower (41–65% less) expression of αSMA mRNA in ACF (Figure 4C) compared to sham CFs (*p* = 0.049). 

### 2.4. MRTF-A Translocates to the Nucleus on Higher Stiffness

**Figure 5:**
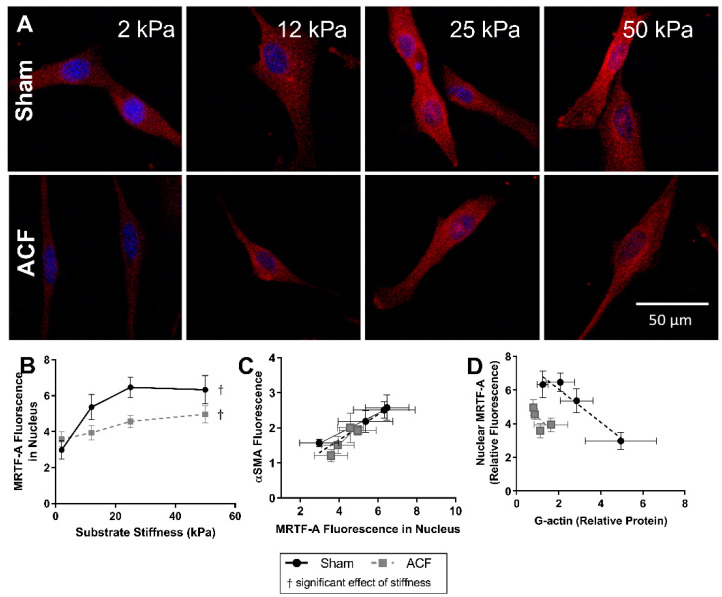
MRTF-A (red) fluorescent localization with DAPI (blue) nuclear counterstain (**A**) and quantification (**B**) in sham and ACF CFs on a range of substrate stiffnesses. MRTF-A nuclear localization is increased with increasing stiffness. (**C**) The relative fluorescence of MRTF-A localized to the nucleus correlates with the relative fluorescence of αSMA fluorescence. (**D**) The relative amount of MRTF-A fluorescence in the nucleus is also inversely related to the amount of G-actin protein quantified. † indicates a significant effect of substrate stiffness.

Since G-actin levels can regulate the localization of MRTF-A and stiffness alters G-actin levels in sham but not ACF CFs (Figure 3C), we determined the effect of stiffness on MRTF-A levels in sham and ACF CFs. Increasing stiffness causes an increase in translocation of MRTF-A to the nucleus as measured by mean fluorescence of MRTF-A staining in both sham and ACF CFs (*p* < 0.001) (Figure 5A,B). With increasing stiffness, there is an increase in nuclear MRTF-A which causes a gradual shift from blue DAPI-stained nucleus, to a more purple nucleus where MRTF-A (red) and DAPI (blue) overlap (Figure 5A). There was a larger increase in nuclear MRTF-A fluorescence in sham compared to a more modest increase in ACF (112% increase between 2 kPa and 25 kPa for sham, *p* < 0.001; 36% increase observed in ACF CF, *p* = 0.0044). The differences at stiffness values were not statistically different between sham and ACF (*p* = 0.25) but the interaction effect, i.e., the observation that sham CFs are more responsive to stiffness, was significant (*p* = 0.0042). Since MRTF-A promotes αSMA expression by binding to the SRF promoter region in the nucleus [25], we looked at the relationship between the amount of MRTF-A fluorescence in the nucleus and total αSMA fluorescence (Figure 5C). For CFs from both ACF and sham rats, total αSMA levels increased approximately linearly with increasing nuclear MRTF-A levels. For both ACF and sham CFs, the relative amount of MRTF-A fluorescence in the nucleus is inversely related to the amount of G-actin protein quantified. The difference between MRTF-A localization in sham and ACF CFs is also influenced by factors other than G-actin levels since for a given level of G-actin, ACF CFs have less nuclear MRTF-A (Figure 5D).

### 2.5. YAP Localizes to the Nucleus More So in Sham CFs Than ACF CFs on Stiff Substrates

**Figure 6:**
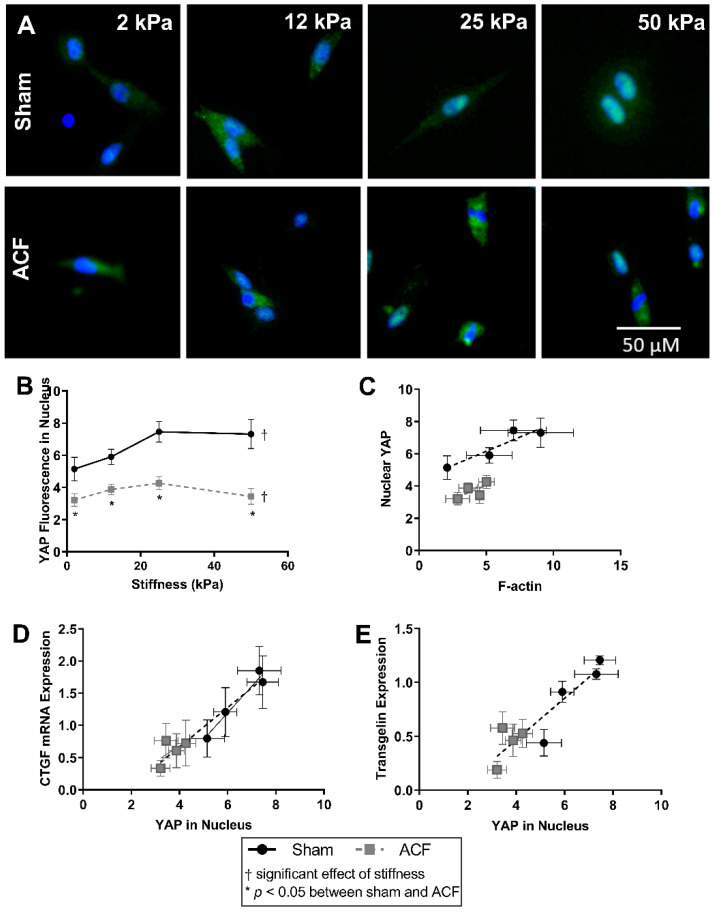
YAP (green) and nuclear (blue) localization using fluorescent staining (**A**) shows a stiffness dependent amount of YAP in the nucleus of Sham CFs, but not ACF. (**B**) Relative YAP fluorescence in the nucleus is lower in ACF overall. (**C**) Nuclear YAP tends to correlate with increased F-actin protein measured in sham CFs, but the amount of nuclear YAP is different between sham and ACF. (**D**,**E**) The amount of YAP in the nucleus correlates with downstream protein production of CTGF (**C**) and transgelin (**E**). * indicates *p* < 0.05 when comparing sham vs. ACF. † indicates a significant effect of substrate stiffness.

Overall, there was a greater amount of YAP fluorescence in the nucleus of sham CFs compared to ACF (37–52% increase compared to ACF, *p* = 0.022) (Figure 6A,B). Increasing stiffness increased the amount of nuclear YAP in sham CFs (a 45% increase in YAP nuclear fluorescence from 2 kPa to 25 kPa, *p* < 0.001). Increased YAP localization in the nucleus can be observed in sham CFs with increasing stiffness by as indicated by a shift from a blue DAPI-stained nucleus to a more turquois nucleus where YAP (green) and DAPI (blue) overlap. Notably, increased stiffness did not significantly increase the amount of YAP nuclear localization in ACF (Figure 6B). For both ACF and sham CFs, the relative amount of YAP fluorescence in the nucleus is positively correlated to the amount of F-actin protein quantified. (Figure 6C).

Nuclear YAP activation directly regulates both CTGF, a profibrotic molecule, and transgelin, an actin bundling protein [26,27]. CTGF expression increases approximately linearly with increasing nuclear YAP (Figure 6D). Relative to CFs from sham hearts, ACF CFs have lower CTGF expression and correlating lower levels of YAP in the nucleus.

Transgelin expression has a similar pattern as CTGF between sham and ACF on different stiffness levels. Transgelin’s regulation is also dependent on YAP activation [27,28]. Figure 6E illustrates an approximately linear relationship between the amount of YAP fluorescence in the nucleus relative to transgelin mRNA expression. This shows that the decreased transgelin expression may be related to decreased levels of nuclear YAP. The inability of elevated stiffness to increase the nuclear localization of YAP helps explain the smaller amounts of CTGF and transgelin mRNA expression observed at all stiffness levels relative to sham.

### 2.6. PPAR-γ Expression Decreases with Increased Stiffness

**Figure 7:**
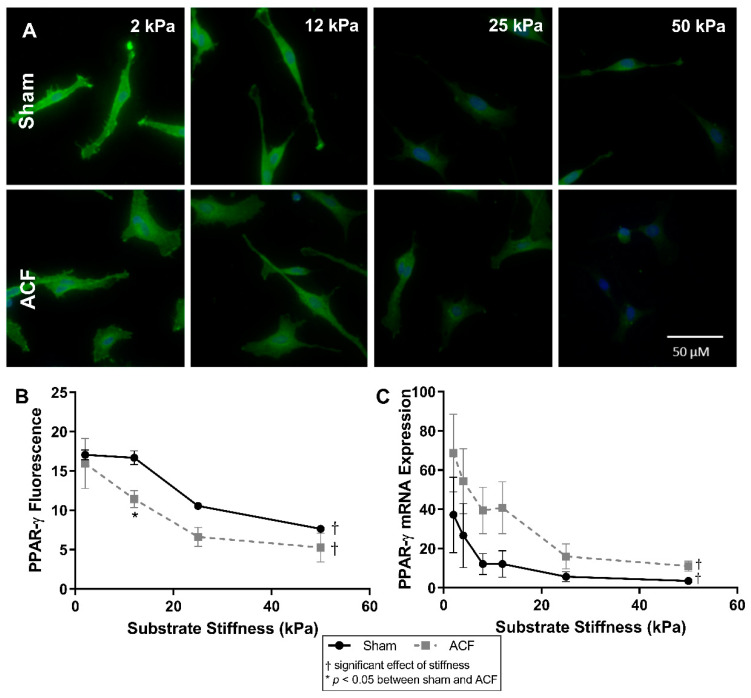
(**A**) PPAR-γ fluorescence (green) with DAPI nuclear counterstain (blue) and quantification (**B**) show a decrease in intensity with increasing stiffness. (**C**) mRNA expression is also inversely related with substrate stiffness. The highest expression and fluorescence and mRNA levels appears on the softest substrates. * indicates *p* < 0.05 when comparing sham vs. ACF. † indicates a significant effect of substrate stiffness.

The expression of peroxisome proliferator-activated receptor gamma (PPAR-γ), an inhibitor of the TGF-β pathway [29,30], has previously been shown to be influenced by stiffness [31,32] so we looked at it here as a potential explanation of the hypofibrotic phenotype of ACF despite increased TGF-β secretion. PPAR-γ expression decreases with increasing stiffness in both sham and ACF as measured by fluorescent staining of PPAR-γ (Figure 7B) and mRNA expression (Figure 7C). In sham CFs there is a 91% decrease in PPAR-γ mRNA (*p* = 0.0074) and a 55% decrease in PPAR-γ fluorescence (*p* < 0.001) between 2 kPa and 50 kPa gels. In ACF CFs there is an 84% decrease in PPAR-γ mRNA (*p* < 0.001) and a 67% decrease in PPAR-γ fluorescence (*p* < 0.001) between 2 kPa and 50 kPa gels. There is not a significant difference at most levels of stiffness between sham and ACF CFs in fluorescence and there is not any statistical difference between sham and ACF PPAR-y mRNA expression at any stiffness (*p* = 0.11).

## 3. Discussion

Here we present data that suggest the importance of substrate stiffness on CF phenotype with increased stiffness promoting cytoskeletal protein production (αSMA and transgelin), increased nuclear translocation of transcriptional activators (YAP and MRTF-A), and decreased expression of transcriptional repressors (PPAR-γ) in normal CFs. In addition, CFs from ACF appear to have a dampened response to stiffness compared to sham CFs. Overall, the data suggest the cytoskeleton and mechanotransduction of stiffness to transcriptional factors may account for some aspects of the hypofibrotic phenotype of ACFs which come from a softer tissue environment in vivo. In our initial report here on the effects of stiffness on CF phenotype, we have focused our attention on only several molecular pathways, especially ones we previously reported to be differentially regulated between ACF and sham fibroblasts [14]. Other mechanisms, especially one shown to be regulated by stiffness in other cell types, merit future investigation.

We are not the first to look at the effect of substrate stiffness on fibroblast phenotype [4,23,33,34,35,36], but aspects of both our study design and results are noteworthy. There are only limited data looking at the effect of stiffness on fibroblasts from the heart [37,38,39,40], and, to our knowledge, there is only one previous report of non-passaged adult CF’s response to substrate stiffness [41], which is discussed in more detail later. The use of non-passaged CFs is an important distinction, since the effect of passaging in culture on fibroblasts to create a more myofibroblast phenotype is well known [42,43]. Relative to cells in vivo, cell cultures are exposed to altered chemical (e.g., higher oxygen tension, which is known to promote myofibroblast activation [44,45]) and biochemical environments (e.g., exposure to elevated PDGF and TGF-β). In addition, cultured cells are typically cultured on substrates that are many orders of magnitude stiffer than they experience in vivo. Prolonged culture on stiffer substrates also leads fibroblasts towards a myofibroblasts phenotype. Comparing our results with non-passaged fibroblasts to others’ results with passaged fibroblasts suggests the importance in using non-passaged fibroblast in our study. Passaged lung fibroblasts activate into myofibroblasts around 20 kPa or higher [19,42] and display a low G/F-actin ratio (~1 in lung fibroblasts on 20 kPa gels compared to 2.4–5.9 ratio in CFs on 25 kPa gels here) [19]. Even on the 50 kPa gels, our CFs do not activate to myofibroblasts during ~1 week of culture. A transition of fibroblasts towards a myofibroblast phenotype would be a significant issue in our study since we are primarily interested in understanding the behavior of CFs from VO hearts where myofibroblasts are not present, and where the CFs are expressing a hypofibrotic phenotype, which contrasts with the profibrotic phenotype exhibited by myofibroblasts.

A more hypofibrotic phenotype is present in sham CFs on softer gels compared to sham CFs on more stiff gels. The stiffnesses (2 kPa) of the polyacrylamide gels we used in our in vitro experiments is less than the ~10 to 15 kPa stiffness sometimes used to describe a normal heart. While we think it could potentially be useful to consider the softest in vitro conditions (2 kPa) as soft relative to a normal heart, one should be cautious when making comparisons between the stiffness of linearly elastic polyacrylamide to the non-linear elastic properties of the heart. We suggest it would be more appropriate to consider our data from the perspective that we explored CFs responses to a wide (2 to 50 kPa or 25-fold) range of stiffnesses in vitro to capture the potential effects of relatively soft and relatively stiff substrates. On soft gels, sham CFs have a less contractile and less polymerized cytoskeleton evident from a lower F/G-actin ratio (Figure 2), decreased αSMA (Figure 4), and reduced transgelin (Figure 1D). Additionally, on soft gels, sham CFs have a profile of transcriptional factors that would discourage a more profibrotic phenotype. For instance, on the softer gels, sham CFs have less MRTF-A and YAP in the nucleus (Figure 5 and Figure 6, respectively), which indicates a decrease in transcriptional activators of fibrogenic programs, and increased PPAR-γ (Figure 7), a transcriptional repressor of TGF-β. Finally, some of the hallmark targets used to describe CF phenotype indicate that the sham CFs on soft gels are relatively hypofibrotic, as indicated by decreased CTGF (Figure 1C) and increased MMP13 expression (Figure 1B). These data together suggest that the decreased stiffness can move normal CFs in the direction of a hypofibrotic phenotype such as that of the CFs taken from ACF.

Overall, ACF CFs tend to display a more hypofibrotic phenotype compared to sham CFs on the same stiffness. ACFs have generally less αSMA and CTGF mRNA expression on most stiffness levels (Figure 1C and Figure 4C). There is a decreased amount of YAP nuclear localization on all stiffness levels (Figure 6B), indicating lower YAP activation. In addition to the decreased αSMA mentioned previously, there is lower transgelin mRNA expression and a decreased reservoir of G-actin, indicating ACFs have a less organized and less contractile cytoskeleton compared to sham CFs. This profile of lower profibrotic molecules and disorganized cytoskeleton is opposite of the phenotype for myofibroblasts. Even on the 50 kPa gels, ACFs have less nuclear YAP, less CTGF expression, and similar amounts of transgelin expression as sham CFs on 2 kPa gels. Taken together, these data imply that ACFs retain elements of a hypofibrotic phenotype in culture even at higher stiffness.

Recently, Gilles et al. reported on their studies of adult mouse cardiac fibroblasts on plastic (stiff substrate) and polyacrylamide gel (soft substrate of 4.5 kPa) [41]. While their goal of establishing in vitro culture conditions that prevented the transitions of CFs to myofibroblasts was different than our goal and they used only cells from healthy hearts, considering their data and our data together provides a more complete view. As we report here, Gilles et al. observed that culture on a less stiff substrate decreased Col1a1, αSMA (Acta2), and CTGF mRNA expression by CFs isolated from healthy hearts. In addition to these markers quantified in both studies, other markers of myofibroblasts quantified in their study (lysyl oxidase and periostin) and our study (F-actin) were reduced by culture on softer substrates. They reported that culture on a soft substrate delayed but did not prevent the transition of cardiac fibroblasts towards myofibroblasts, which again emphasizes the importance of using primary CFs shortly after isolation in our work.

The data summarized above strongly support the notion that decreasing substrate stiffness can push CFs towards a hypofibrotic phenotype and support our notion that the ~50% reduction in stiffness seen in volume hearts [15] contributes to hypofibrotic nature of the CFs isolated from these hearts [14]. A potential objection to this view is that since CFs respond to their current stiffness in culture, any measurements made in vitro will reflect their current substrate stiffness and not what they experienced previously in vivo. While acknowledging this potential objection, we do not believe it is valid. Others have shown that cells exhibit “mechanical memory”, that is, cells cultured for extended time on substrates of various stiffness are ingrained with aspects of the phenotypic changes brought on by that substrate stiffness even after the cells are moved to a new substrate with a different stiffness. One example of mechanical memory is that YAP activation of MSCs plated on a stiff substrate and then plated on a softer substrate is dependent on the amount of time spent on the original stiff substrate [32]. A similar phenomenon was also observed in lung fibroblasts cultured on soft substrates for 3 weeks, which displayed a dampened myofibroblast activation when plated on stiff substrates [42]. The mechanical memory of cells to the stiffness that they were exposed to chronically in the past, coupled with our and others’ observations that cells acutely adapt to their current substrate stiffness, explains our observation that ACF CFs have a hypofibrotic phenotype in vitro, even when grown on stiff substrates, i.e., these non-passaged CFs retain aspects of their soft in vivo phenotype in culture.

Considering the potential in vivo significance of these observations, our notion that reduced substrate stiffness pushes CFs towards a hypofibrotic phenotype suggests that a viscous cycle potentially occurring during VO heart failure with the reduction in the stiffness of the heart pushes CFs towards a hypofibrotic phenotype, and the reduced ECM deposition and increased ECM degradation associated with the hypofibrotic phenotype, resulting in further decreases in substrate stiffness. While significance of this potential vicious cycle in the pathophysiology of heart failure is not known, this model is consistent with the available data and suggests future lines of inquiry.

These results point to the role stiffness plays in modulating CF phenotype. Here we show that normal CFs behave more like hypofibrotic CFs on soft substrates and that CFs from ACF have a dampened response to stiffness. Our observations that ACFs have approximately half the tissue modulus compared to sham [15], that CFs isolated from ACF have a more hypofibrotic phenotype compared to sham CFs [14], and here that a more hypofibrotic phenotype results from exposure to decreased substrate modulus in CFs, suggest the reduced stiffness in ACF hearts may cause CFs to adopt a hypofibrotic phenotype. In addition, our previous observations that ACFs are primed in an environment with decreased tissue modulus [15] yet retain a hypofibrotic phenotype even in culture on stiff plastic [14]), coupled with our observations here that ACF CFs have a dampened response to stiffness compared to sham CFs, is consistent with the notion of mechanical memory.

## 4. Materials and Methods

### 4.1. Animals

Male Sprague Dawley (~200 g, Envigo, Indianapolis, IN, USA) were kept in temperature and humidity-controlled housing, with free access to standard chow and water, and with a 12-hour light/dark cycle. Studies conformed to the principles of the National Institutes of Health “Guide for the Care and Use of Laboratory Animals,” (NIH publication No. 85-12, revised 1996). The protocol was approved by the Institutional Animal Care and Use Committee of The Research Institute at Nationwide Children’s Hospital (protocol #AR09-00053, approval date: 2/4/2016). Age- and weight-matched animals were used for sham and aortocaval fistula (ACF) surgeries (*n* = 3–7). ACF surgeries have been described previously [10,46], briefly, animals were anaesthetized with ~2% isoflurane, an abdominal incision was made, the abdominal aorta and inferior vena cava were exposed with blunt dissection and an 18-gauge needle was inserted into the shared wall of the vessels. The opening on the inferior vena cava was closed with either cyanoacrylate glue or purse string sutures. Arterial mixing in the vena cava was visually confirmed and the abdomen closed with sutures. The sham surgery is similar, where the vessels are exposed by blunt dissection and the abdomen is closed with sutures. Buprenex was given for pain at 24 and 72 h post-operatively and as needed. VO was confirmed 4 weeks after surgery via echocardiography, with a left ventricular end diastolic diameter (LVEDD) of at least 8 mm.

### 4.2. Cardiac Fibroblasts

We used a protocol to isolated primary adult CFs from rats as described previously [14]. Briefly, CFs are isolated from LVs of 4-week post ACF or sham rats by enzymatic digestion (80 U collagenase type-2 and 0.1% trypsin) and plated in 10% fetal bovine serum–Dulbecco’s modified essential media. Each cell solution, from an individual animal, was plated on a range of polyacrylamide gels (Matrigen, Irvine, CA, USA) pre-coated with a solution of 10 µg/mL type I rat tail collagen (BD) in 0.02 N acetic acid. Approximately one hour later, media were replaced with 10% FBS DMEM with 1.0 g/L glucose. Cells were gently washed several times with warm PBS 24 h after isolation and fresh media were replaced until cells reached a confluence of ~60–80%.

### 4.3. Fluorescent Staining

Cells were fixed in 4% paraformaldehyde in PBS solution for half an hour. Fixed cells were then permeabilized with 0.03% Triton-X and blocked in BSA and goat serum or fish block (for MRTF-A staining) for 1 h. Primary antibodies were incubated overnight at 4 °C with gentle agitation (anti-MRTF-A, Santa Cruz, Dallas, TX, USA, sc-21558; anti-YAP, Cell Signaling, Danvers, MA, USA 14074; anti-PPARγ, Pierce, Waltham, MA, USA, PA3-821A; anti-αSMA, Sigma, St. Louis, MO, USA, a2547). Primary antibodies were washed with PBS several times prior to incubation with fluorescent secondary antibodies. Counterstains were done with DAPI, to stain nuclei, and TRITC conjugated phalloidin, to stain F-actin (Millipore, Burlington, MA, USA, FAK100) or Alexa Fluor 488-conjugated DNAse-I (Thermo Scientific, Waltham, MA, USA), to preferentially stain G-actin (Thermo Fisher Scientific, Waltham, MA, USA). Fluorescent micrographs were taken with an Olympus IX51 microscope (to visualize YAP, MRTF-A, and PPAR-γ) or Zeiss 710 confocal microscope (to visualize F-actin, G-actin, αSMA). Fluorescent intensity was quantified on at least 50 cells per condition, *n* = 3–4 animal replicates, using ImageJ. DAPI counterstains were used to create masks to identify nuclear regions for YAP and MRTF-A localization for quantitation.

### 4.4. Real Time Quantitative PCR

CFs (from *n* = 7 animal replicates) were lysed in TRIzol extraction reagent, scraped, and sonicated. RNA was extracted with chloroform and centrifuged. The aqueous phase was purified using Qiagen RNeasy Mini Kit (Qiagen, Germantown, MD, USA) and RNA concentration was determined by a NanoDrop 2000 (Thermo Scientific, Waltham, MA, USA) spectrophotometer. Maxima First Strand cDNA Synthesis Kit (Thermo Scientific, Waltham, MA, USA) was used to reverse transcribe RNA. Equivalent amounts of cDNA were amplified in duplicate with Maxima Probe qPCR master mix (Thermo scientific, Waltham, MA, USA) and Roche Universal Probe and primer pairs for the target genes (See Table 1 for sequences). Forty amplification cycles were carried out using an Eppendorf MasterCylcler-ep Realplex thermocycler. Relative expression was determined by using the 2^−ΔCt^ method, normalizing to the mean of the housekeeping genes Rpl13a and LDHA.

### 4.5. G-Actin and F-Actin Immunoblot

The ratio of F-actin and G-actin were analyzed using a kit from Cytoskeleton (Denver, CO, USA) and according to the manufacturer provided protocol. Briefly, cell lysates (*n* = 4, animal replicates) were scraped and collected with F-actin stabilization lysis buffer provided in the kit. Lysates were briefly sonicated and centrifuged at 350× *g* to pellet unbroken cells and debris and the supernatant was centrifuged at 100,000× *g* at 37 °C for 1 h to pellet F-actin. The supernatant contained G-actin, and the F-actin pellet was suspended in an equal volume to the supernatant with F-actin depolymerizing buffer provided in the kit. The G-actin and F-actin solutions were prepared in SDS loading buffer and run through an SDS-PAGE gel in parallel. The supernatant (G-actin) and pellet (F-actin) from a single sample were always run on the same gel to make accurate comparisons for F/G-actin ratio values. Due to the high number of samples, some replicates had to be run on a separate gel. Specifically, 2 of the 4 replicates for sham and ACF in the 2 kPa condition had to be run on a separate gel. Gels were transferred to a PVDF membrane, blocked in 5% milk, and incubated with a rabbit anti-pan-actin antibody (at 1:500, 43 kDa) provided in the kit. The blots were then incubated with an anti-rabbit horseradish peroxidase (HRP) secondary antibody (1:1500), and F/G-actin ratios were determined by chemiluminescence densitometry. The blots were incubated with stripping buffer to remove antibodies and then incubated with secondary HRP antibody to verify the removal of the primary anti-pan actin antibody with an absence of signal. Blots were then incubated with total ERK1/2 (ERK1, Santa Cruz, sc93, 1:5000, ERK 2, Santa Cruz, sc154, 1:5000)) and used to normalize protein loading to find relative amount of G-actin and F-actin protein.

### 4.6. Statistics

Results are reported as mean ± standard error of the mean. Statistical analysis was performed with a two-way analysis of variance and differences between conditions were assessed by a post-hoc Tukey’s test. A *p*-value less than 0.05 is reported as statistically significant.

## Figures and Tables

**Table 1:** PCR Primer Sequences.

Target	Gene	Accession Number	Forward Primer Sequence	Reverse Primer Sequence
α-smooth muscle actin	ACTA2	NM_031004.2	TGCCATGTATGTGGCTATTCA	ACCAGTTGTACGTCCAGAAGC
collagen type-1 α-1	COL1A1	NM_053304	TCTGGTCTCCAGGGTCCTC	GTCCATCTTTGCCAGGAGAA
connective tissue growth factor	CTGF	NM_022266	GCTGACCTAGAGGAAAACATTAAGA	CCGGTAGGTCTTCACACTGG
lactate dehydrogenase A	LDHA	NM_017025.1	GATGATGGATCTTCAGCATGG	GCTTGGAGTTTGCAGTCACA
MMP-13	MMP13	NM_133530	GGACAAGCAGCTCCAAAGG	GGTCCAGACCGAGGGAGT
PPAR-γ	Pparg	NM_013124.3	GGTGAAACTCTGGGAGATCCT	AATGGCATCTCTGTGTCAACC
ribosomal protein L13A	Rpl13a	NM_173340.2	CCCTCCACCCTATGACAAGA	GGTACTTCCACCCGACCTC
transgelin (SM22)	TAGLN	NM_031549.2	AGTGTGGCCCTGATGTGG	TCACCAACTTGCTCAGAATCA

## Data Availability

Data is contained within the article or may be obtained upon request.

## Abbreviations

αSMA	Alpha Smooth Muscle Actin
ACF	Aortocaval Fistula
CF	Cardiac Fibroblast
CTGF	Connective Tissue Growth Factor
DAPI	4′,6-diamidino-2-phenylindole
DMEM	Dulbecco’s Modified Eagle Medium
ERK	Extracellular signal-related protein kinase
FBS	Fetal bovine serum
HF	Heart Failure
HRP	Horseradish peroxidase
LVEDD	Left ventricular end-diastolic diameter
MMP	Matrix Metalloproteinase
MRTF-A	Myocardin-Related Transcription Factor A
PAGE	Polyacrylamide gel electrophoresis
PDGF	Platelet-derived growth factor
PPARγ	Peroxisome proliferator-activated receptor gamma
PVDF	Polyvinylidene difluoride
SDS	Sodium Dodecyl Sulphate
SRF	Serum response factor
TEAD	Transcriptional enhancer factor domain
TGF-β	Transforming Growth Factor Beta
VO	Volume Overload
YAP	Yes-Associated Protein
